# Hemi-Synthesis and Anti-Oomycete Activity of Analogues of Isocordoin

**DOI:** 10.3390/molecules22060968

**Published:** 2017-06-10

**Authors:** Beatriz Escobar, Iván Montenegro, Joan Villena, Enrique Werner, Patricio Godoy, Yusser Olguín, Alejandro Madrid

**Affiliations:** 1 Departamento de Química, Facultad de Ciencias Naturales y Exactas, Universidad de Playa Ancha, Avda. Leopoldo Carvallo 270, Playa Ancha, Valparaíso 2340000, Chile; escobarbeatriz25@gmail.com; 2 Escuela de Obstetricia y Puericultura, Facultad de medicina, Campus de la Salud, Universidad de Valparaíso, Angamos 655, Reñaca, Viña del Mar 2520000, Chile; ivan.montenegro@uv.cl; 3 Centro de Investigaciones Biomédicas (CIB), Escuela de Medicina, Universidad de Valparaíso, Av. Hontaneda Nº 2664, Valparaíso 2340000, Chile; juan.villena@uv.cl; 4 Departamento De Ciencias Básicas, Campus Fernando May Universidad del Biobío. Avda. Andrés Bello s/n casilla 447, Chillán 3780000, Chile; ewerner@ubiobio.cl; 5 Instituto de Microbiología Clínica, Facultad de Medicina, Universidad Austral de Chile, Los Laureles s/n, Isla Teja, Valdivia 5090000, Chile; patricio.godoy@uach.cl; 6 Center for Integrative Medicine and Innovative Science (CIMIS), Facultad de Medicina, Universidad Andrés Bello, Santiago 8320000, Chile; yusser.olguin@unab.cl

**Keywords:** isocordoin, oxyalkyl derivates, anti-oomycete activity, *Saprolegnia* sp.

## Abstract

An efficient synthesis of a series of 4′-oxyalkyl-isocordoin analogues (**2**–**8**) is reported for the first time. Their structures were confirmed by ^1^H-NMR, ^13^C-NMR, and HRMS. Their anti-oomycete activity was evaluated by mycelium and spores inhibition assay against two selected pathogenic oomycetes strains: *Saprolegnia parasitica* and *Saprolegnia australis*. The entire series of isocordoin derivatives (except compound **7**) showed high inhibitory activity against these oomycete strains. Among them, compound **2** exhibited strong activity, with minimum inhibitory concentration (MIC) and minimum oomyceticidal concentration (MOC) values of 50 µg/mL and 75 µg/mL, respectively. The results showed that 4′-oxyalkylated analogues of isocordoin could be potential anti-oomycete agents.

## 1. Introduction

Chalcones bearing prenyl groups are an abundant subclass of naturally occurring chalcones [1]. The biosynthesis of *C*-prenyl chalcones involves the coupling of products from the shikimic and mevalonic acid pathways. The latter produces the prenyl group, while the former produces the aromatic compound. The two products are linked together by prenyl tranferase enzymes [2]. Almost 80% of these molecules are monoprenylated and the remaining 20% consist of di and/or triprenyl derivatives. *C*-prenylation takes place more frequently on ring A at positions 3′ and/or 5′, as well as 3 and/or 5 [3]. Prenylated chalcones have been isolated from plants mainly belonging to the families of Leguminosae and Moraceae [4]. This class of compounds has been found to display a variety of biological activities that are anticarcinogenic, antiprotozoal, larvicidal, anti-inflammatory, antibacterial, antioxidant, and antifungal [5,6]. A representative example of naturally occurring bioactive *C*-prenylated chalcones, isocordoin (**1**), was isolated from *Lonchocarpus* species [7,8] or *Aeschynomene fascicularis* [9]. Isocordoin has been found to have a range of interesting biological properties in vitro that may have therapeutic and industrial utilities including antiprotozoal (for treatment of *Leishmania mexicana*, *L. braziliensis*, *L. amazonensis* and *Trypanosoma cruzi*) [10,11], insect antifeedant (for treating lepidopterous larvae of *Spodoptera littoralis* and *S. exempta*, which are considered one of the most destructive pests of crops) [12] and a vasorelaxant effect [13], as well as a potential “broad-spectrum” anticancer agent (applicable to both oral-laryngeal and prostate cancers) [9,10,14]. These bioactivities have stimulated the interest of researchers in the preparation of isocordoin. The synthesis of **1** via the Claisen–Schmidt condensation was obtained in unsatisfactory yields [15,16]. In contrast, the enzymatic prenylation of 2′,4′-dihydroxychalcone with cell cultures of *Morus nigra* biosynthetically produced isocordoin in very high yield [16,17]. Recent works have explored the introduction of isoprenoids into the structure of chalcones to obtain more active hybrid compounds [18], or even with new biological properties for the development of novel products either in medicine or in agrochemistry. In this paper, a series of 4-oxyalkyl-isocordoin analogues **2**–**8** were synthesized and evaluated for their anti-oomycete activity against *Saprolegnia parasitica* and *S. australis*. 

## 2. Results and Discussion

### 2.1. Synthesis and Characterization 

**Scheme 1:**
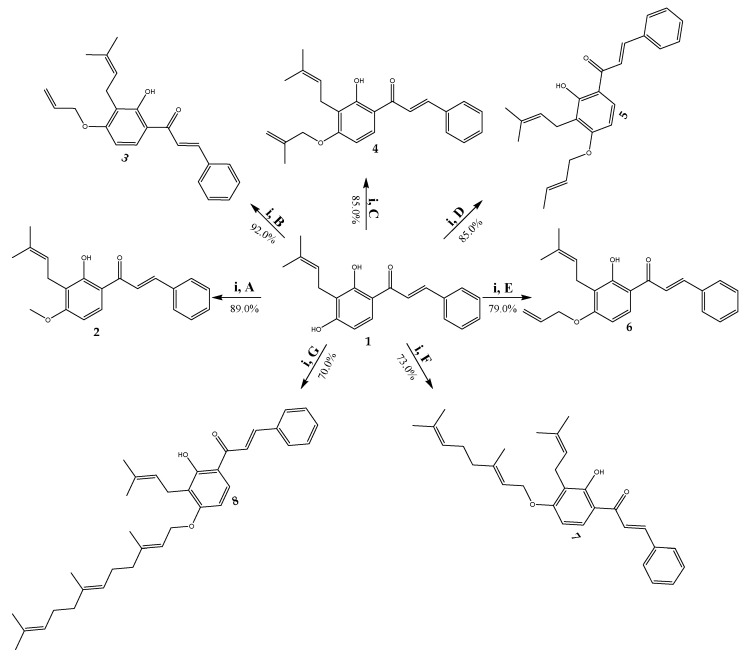
Synthesis of 4-oxyalkyl-isocordoin derivatives **2**–**8**. Reagents and conditions: (i) K_2_CO_3_/CH_3_CN, reflux at 80 °C for 4 h.; (**A**) CH_3_I; (**B**) allyl bromide; (**C**) 2-methyl-1-propenyl bromide; (**D**) crotyl bromide; (**E**) prenyl bromide; (**F**) geranyl bromide; (**G**) farnesyl bromide.

One naturally occurring chalcone, **1**, and seven oxyalkyl derivates, **2**–**8**, were synthesized. The synthetic strategies applied for the synthesis of new chalcones are outlined in Scheme 1 [19,20].

Isocordoin (**1**) was purified in analogy to the procedures described in [21,22] with small variations from the resinous exudate of *Adesmia balsamica*. The targeted compounds **2**–**8** were easily attained in high yields (70–92%) through nucleophilic substitution of isocordoin with the appropriate alkyl halide in acetonitrile. This reaction resulted in the exclusive formation of the 4-oxyalkyl-derivative, due to steric hindrance caused by the the prenyl chain in position 3′ of the aromatic ring A [23,24] and the influence exerted by the intramolecular hydrogen bond present in the isocordoin [25].

The structures of compounds **1**–**8** were established on the basis of IR, NMR, and HR-MS techniques. ^13^C-NMR assignments were achieved by 2D heteronuclear single quantum correlation (HSQC) and heteronuclear multiple bond correlation (HMBC) experiments. 

**Figure 1:**
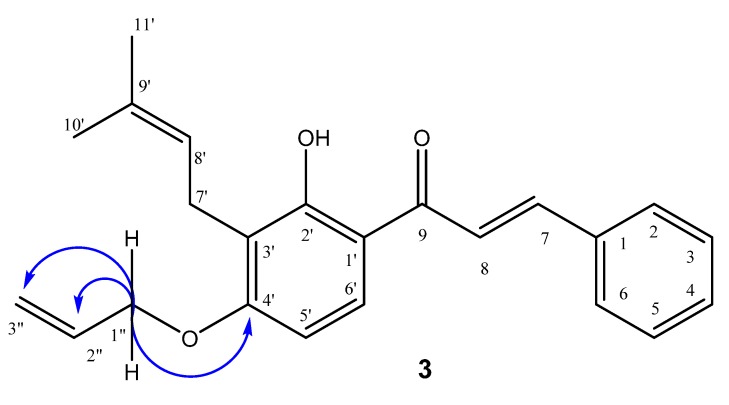
The most important correlations shown by 2D ^1^H-^13^C HMBC, compound **3**.

The IR, NMR, and MS data of compound **1** concurred with those reported for the known chalcone, commonly named as isocordoin, previously reported from *Lonchocarpus xuul* [8]. The ^1^H spectrum of 4′-oxyalkyl-isocordoin derivatives **2**–**8** showed a number of signals that correspond to an isocordoin-type structure. The spectra of these compounds showed two trans-olefinic protons at δ = 7.89 and 7.60 ppm (d, *J* = 15.4 Hz) readily assigned to those of the 8 and 7 position, respectively; two aromatic protons at ortho positions (d, *J* = 9.1 Hz at δ = 6.48 and 7.77 ppm); and the presence of a prenyl group (d, *J* = 7.1 Hz, 2H at δ = 3.39–3.40 ppm; m, 1H at δ = 5.25 ppm; and two singlets, 3H each, at δ = 1.68 and 1.80 ppm), and two multiplets at δ = 7.65 ppm (2H) and 7.42 ppm (3H), corresponding to the five protons of a unsubstituted aromatic ring. The ^1^H-NMR spectrum of **2**–**8** showed a chelated hydroxyl-proton signal at δ 13.39–13.37 ppm, together with a chelated carbonyl-carbon resonance at δ = 192.1 ppm, were consistent with the selectivity of the *O*-alkylation at position 4′ of isocordoin. The compound **2**, obtained as an amorphous bright yellow solid, proved to be identical to derricin, previously reported from *Lonchocarpus sericeus* [26]. For 4′-oxyalkyl-isocordoin derivatives **3**–**8** the ^1^H-NMR spectrum revealed resonances at δ = 4.54–4.65 ppm (d, 2H) ascribed to O-CH_2_ protons of alkoxy chain linking 4′-OH-chalcone ring. This is characteristic of allylic chains on aromatic rings, resulting by the alkylation reaction. For example, the ^1^H-NMR spectrum of compound **3** showed the signal at 4.65 ppm (d, *J* = 5.1 Hz, 2H, H-1′′) correlated (by 2D HSQC) with a carbon atom at δ 65.4 ppm (C-1″), indicating the coupling point between the allyl fragment and the aromatic hydroxyl group. These data also were corroborated by 2D HMBC correlations, where H-1″ showed heteronuclear *^3^J* correlations with the carbons signal at δ = 117.6 (C-3′′) and 162.6 (C-4′) ppm. Heteronuclear *^2^J* correlations were also observed at δ 132.7 (C-2″) (Figure 1).

### 2.2. Anti-Oomycete Activity Assays of Compounds ***1**–**8***

In this study, isocordoin (**1**) and a series of seven oxyalkylated derivatives (**2**–**8**) were screened for minimum inhibitory concentration (MIC) values (Table 1).

Among them, isocordoin (**1**) and compound **7** were considered to have no inhibition effect against *Saprolegnia* with MIC values ≥ 200.0 µg/mL, while the other six compounds showed anti-oomycete activities with MIC ≤ 150.0 µg/mL, in which derricin (**2**) and compound **3** had the most promising anti-*Saprolegnia* activities with MIC values of 50 and 75 µg/mL, respectively.

Table 2 lists the minimum oomyceticidal concentration (MOC) values of compounds **1**–**8** against *Saprolegnia* spores at 72 h. The results reveal that derricin (**2**) showed significant anti-oomycete activity with an MOC value of 75 µg/mL, followed by compound **3** with a value of 100 µg/mL.

Table 3 lists the mycelial growth inhibition (MIG) percentage values of compounds **1**–**8** against *Saprolegnia* mycelium at 48 h.

The growth inhibition rate of *Saprolegnia* after 48 h of exposure to derricin (**2**) to was partially affected by the concentration of the chemical. However, it was more than twice as effective as bronopol, a broad-spectrum biocide, that has been used as an effective and economically acceptable alternative in the treatment and control of *Saprolegnia* sp. [20]. The inhibition of *S. parasitica* and *S. australis* was 52% and 58%, respectively, with concentrations of derricin at 200 µg/mL.

The results of this research provides evidence that lipophilicity is an important characteristic of anti-oomycete agents, such as ketoconazole and clotrimazole [27,28,29,30], and it is also evident that drugs with oxyalkyl chains on the A-ring contribute to an increase in anti-oomycete activity [20,31], which has proven effectiveness in the treatment of Saprolegniasis.

## 3. Materials and Methods 

### 3.1. General

All chemicals were obtained from Aldrich (St. Louis, MO, USA) and were used without further purification. A detailed description of conditions used to register Fourier transform infrared (FT-IR) spectra, high resolution mass spectra, and ^1^H, ^13^C, ^13^C DEPT-135, gs-2D Heteronuclear Single Quantum Coherence (HSQC) and gs-2D HMBC spectra has been given elsewhere [32]. Column chromatography (CC) was performed with silica gel 60 from Merck (Darmstadt, Germany), and thin layer chromatography (TLC) was carried out on precoated silica plates F-254 from Merck (Darmstadt, Germany). Melting points were determined on an electrothermal instrument and are uncorrected. 

### 3.2. Plant Material

*Adesmia balsamica* were collected in Melosillas, Casablanca, Chile, in October 2016. A voucher specimen was deposited at the Valparaíso (VALP) Herbarium, Department of Biology, Universidad de Playa Ancha, Valparaíso, Chile and was identified by comparison with the herbarium specimen (collection number 1899).

### 3.3. Isolation and Characterization of Isocordoin *(**1**)*

The fresh plant (1.0 kg) extracted with cold dichloromethane (5 L) at room temperature for 40 s. The organic extract was concentrated in a rotatory evaporator to give a residue of 120.5 g of resin. Pure isocordoin (**1**) was isolated to obtain 166.5 mg of a solid yellow powder. Its structure was established by comparison of their spectroscopic data (^1^H and ^13^C-NMR and HRMS) with those in the literature [8]. 

### 3.4. General Procedure for the Synthesis of 4-Oxyalkyl-isocordoin Analogues *(**2**–**8**)*

A solution of isocordoin (**1**) (5 mmol), alkyl halide (6 mmol), and K_2_CO_3_ (6.5 mmol) in anhydrous acetonitrile (3 mL) was refluxed for 4 h at 80 °C. After the reaction, the mixture was distilled to remove the solvent. The residue was poured into ice water (40 mL), and then filtered. The resulting filtrate was extracted three times with ethylacetate. The resulting organic phase was dried over anhydrous magnesium sulfate, and distilled in vacuo to remove the solvent to provide the target compounds **2**–**8**. 

*(2E)-1-[2-Hydroxy-4-methoxy-3-(3-methylbut-2-en-1-yl)phenyl]-3-phenylprop-2-en-1-one* (**2**). Yellow solid. Yield: 89.0%. Spectroscopic data and physical properties of compound **2** were consistent with those reported in the literature [26]. 

*(2E)-1-[4-(Allyloxy)-2-hydroxy-3-(3-methylbut-2-en-1-yl)phenyl]-3-phenylprop-2-en-1-one* (**3**). Yellow solid. Yield: 92.0%, melting point: 93–96 °C. IR (KBr, cm^−1^): 3024, 2895, 1647, 1577, 1298, 1195, and 1134. ^1^H-NMR (400 MHz, CDCl_3_): δ 13.37 (s, 1H, 2′-OH), 7.88 (d, *J* = 15.4 Hz, 1H, H-8); 7.77 (d, *J* = 9.1 Hz, 1H, H-6′); 7.65 (m, 2H, H-2 and H-6); 7.60 (d, *J* = 15.4 Hz, 1H, H-7); 7.42 (m, 3H, H-3, H-4 and H-5); 6.48 (d, *J* = 9.0 Hz, 1H, H-5′); 6.07 (m, 1H, H-2′′); 5.45 (m, 1H, H-3′′b); 5.31 (m, 1H, H-3′′a); 5.26 (m, 1H, H-8′); 4.65 (d, J = 5.1 Hz, 2H, H-1′′); 3.40 (d, *J* = 7,1 Hz, 1H, H-7′); 1.80 (s, 3H, H-10′); 1.68 (s, 3H, H-11′). ^13^C-NMR (100 MHz, CDCl_3_): δ 192.4 (C-9); 163.2 (C-2′); 162.4 (C-4′); 144.1 (C-7); 134.8 (C-1); 132.7 (C-2′′); 131.8 (C-6′); 130.5 (C-9′); 129.1 (C-4); 129.0 (C-2 y C-6); 128.5 (C-3 y C-5); 121.9 (C-8′); 120.6 (C-8); 118.4 (C-3′); 117.6 (C-3′′); 114.6 (C-1′); 103.2 (C-5′); 65.4 (C-1′′); 25.8 (C-11′); 21.8 (C-7′); 17.9 (C-10′). MS: M + H ion *m*/*z* 349.4432 (C_23_H_24_O_3_: 348.4348).

*(2E)-1-[4-[(2-Methylprop-2-en-1-yl)oxy]-2-hydroxy-3-(3-methylbut-2-en-1-yl)phenyl]-3-phenylprop-2-en-1-one* (**4**). Yellow solid. Yield: 85.0%, melting point: 99–101 °C. IR (KBr, cm^−1^): 2958, 1635, 1519, 1243, and 1131. ^1^H-NMR (400 MHz, CDCl_3_): δ 13.39 (s, 1H, 2′-OH), 7.87(d, *J* = 15.5 Hz, 1H, H-8); 7.77 (d, *J* = 9.0 Hz, 1H, H-6′); 7.65 (m, 2H, H-2 and H-6); 7.60 (d, *J* = 15.4 Hz, 1H, H-7); 7.42 (m, 3H, H-3, H-4 and H-5); 6.48 (d, *J* = 9.0 Hz, 1H, H-5′); 5.26 (m, 1H, H-2′′); 5.11 (s, 1H, H-3b′′); 5.01 (s, 1H, H-3a′′); 4.54 (s, 2H, H-1′′); 3.43 (d, *J* = 7.1Hz, 1H, H-7′); 1.84 (s, 3H, H-4′′); 1.80 (s, 3H, H-10′); 1.68 (s, 3H, H-11′). ^13^C-NMR (100 MHz, CDCl_3_ ): δ 192.2 (C-9); 163.2 (C-2′); 162.4 (C-4′); 144.1 (C-7); 140.3 (C-2′′); 134.9(C-1); 131.8 (C-6′); 130.5 (C-9′); 129.1 (C-4); 129.0 (C-2 and C-6); 128.5 (C-3 and C-5); 122.0 (C-8′); 120.6 (C-8); 117.8 (C-3′); 114.6 (C-1′); 113.0 (C-3′′); 103.2 (C-5′); 71.9 (C-1′′); 25.8 (C-11′); 21.8 (C-7′); 19.3 (C-4′′); 17.9 (C-10′). MS: M + H ion *m*/*z* 363.4702 (C_24_ H_26_O_3_: 362.4616).

*(2E)-1-[4-(Crotyloxy)-2-hydroxy-3-(3-methylbut-2-en-1-yl)phenyl]-3-phenylprop-2-en-1-one* (**5**). Yellow solid. Yield: 85.0%, melting point: 93–94 °C. IR (KBr, cm^−1^): 2958, 1635, 1519, 1243, and 1131. ^1^H-NMR (400 MHz, CDCl_3_): δ 13.37 (s, 1H, 2′-OH), 7.87 (d, *J* = 15.5 Hz, 1H, H-8); 7.77 (d, *J* = 9.1 Hz, 1H, H-6′); 7.65 (m, 2H, H-2 and H-6); 7.60 (d, *J* = 15.4 Hz, 1H, H-7); 7.42 (m, 3H, H-3, H-4 and H-5); 6.49 (d, *J* = 9,0 Hz, 1H, H-5′); 5.87 (m, 1H, H-2′′); 5.82 (m, 1H, H-3′′); 5.26 (m, 1H, H-8′); 4.57 (d, J = 5.9 Hz, 2H, H-1′′); 3.40 (d, *J* = 7.2 Hz, 1H, H-7′); 1.80 (s, 3H, H-10′); 1.77 (s, 3H, H-4′′); 1.68 (s, 3H, H-11′). ^13^C-NMR (100 MHz, CDCl_3_): δ 192.2 (C-9); 163.2 (C-2′); 162.6 (C-4′); 144.0 (C-7); 134.9 (C-1); 131.7 (C-6′); 130.5 (C-9′); 130.3 (C-4); 129.1 (C-2′′); 129.0 (C-2 and C-6); 128.5 (C-3 and C-5); 125.6 (C-3′′); 122.0 (C-8′); 120.7 (C-8); 118.0 (C-3′); 117.8 (C-3′); 115.6 (C-1′); 103.3 (C-5′); 69.0 (C-1′′); 25.8 (C-11′); 21.8 (C-7′); 17.9 (C-10′); 17.8 (C-4′′). MS: M + H ion *m*/*z* 363.4702 (C_24_H_26_O_3_: 362.4614).

*(2E)-1-[4-(Prenyloxy)-2-hydroxy-3-(3-methylbut-2-en-1-yl)phenyl]-3-phenylprop-2-en-1-one* (**6**). Yellow solid. Yield: 79.0%, melting point: 106–108 °C. IR (KBr, cm^−1^): 2959, 1631, 1519, 1374, 1211, 1147, and 990. ^1^H-NMR (400 MHz, CDCl_3_): δ 13.37 (s, 1H, 2′-OH), 7.87 (d, *J* = 15.5 Hz, 1H, H-8); 7.77 (d, *J* = 9.1 Hz, 1H, H-6′); 7.65 (m, 2H, H-2 and H-6); 7.60 (d, *J* = 15.4 Hz, 1H, H-7); 7.42 (m, 3H, H-3, H-4 and H-5); 6.49 (d, *J* = 9.0 Hz, 1H, H-5′); 5.48 (m, 1H, H-2′′); 5.25 (m, 1H, H-8′); 4,62 (d, *J* = 6.6 Hz, 2H, H-1′′); 3.39 (d, *J* = 7.2 Hz, 1H, H-7′); 1.80 (s, 6H, H-4′′ and H-10′); 1.75 (s, 3H, H-5′′); 1.68 (s, 3H, H-11′). ^13^C-NMR (100 MHz, CDCl_3_): δ 192.1 (C-9); 163.2 (C-2′); 162.8 (C-4′); 144.0 (C-7); 138.1 (C-3′′); 134.9 (C-1); 131.7 (C-6′); 130.5 (C-9′); 129.1 (C-4); 129.0 (C-2 and C-6); 128,5 (C-3 and C-5); 122.1 (C-8′); 120.7 (C-8); 119.5 (C-2′′) 117.9 (C-3′); 114.5 (C-1′); 103.3 (C-5′); 65.3 (C-1′′); 25.8 (C-4′′); 25.7 (C-11′); 21.8 (C-7′); 18.3 (C-5′′); 17.8 (C-10′). MS: M + H ion *m*/*z* 377.4972 (C_25_H_28_O_3_: 376.4880).

*(2E)-1-[4-(Geranyloxy)-2-hydroxy-3-(3-methylbut-2-en-1-yl)phenyl]-3-phenylprop-2-en-1-one* (**7**). Yellow solid. Yield: 73.0%, melting point: 78–80 °C. IR (KBr, cm^−1^): 2940, 2865, 1631, 1530, 1512, 1338, 1241, 1129, 817. ^1^H-NMR (400 MHz, CDCl_3_): δ 13.37 (s, 1H, 2′-OH), 7,87 (d, *J* = 15.5 Hz, 1H, H-8); 7.77 (d, *J* = 9.1 Hz, 1H, H-6′); 7.65 (m, 2H, H-2 and H-6); 7.60 (d, *J* = 15.4 Hz, 1H, H-7); 7.42 (m, 3H, H-3, H-4 and H-5); 6.49 (d, *J* = 9.0 Hz, 1H, H-5′); 5.48 (m, 1H, H-2′′); 5.25 (m, 1H, H-8′); 5.08 (m, 1H, H-6′′); 4.65 (d, *J* = 6.4 Hz,2H, H-1′′); 3.39 (d, *J* = 7.2 Hz, 1H, H-7′); 2.10 (m, 4H, H-4′′ and H-5′′); 1.80 (s, 3H, H-10′); 1.75 (s, 3H, H-9′′); 1.68 (s, 3H, H-8′′); 1.61 (s, 3H, H-11′). ^13^C-NMR (100 MHz, CDCl_3_): δ 191.1 (C-9); 163.1 (C-2′); 162.8 (C-4′); 144.0 (C-7); 141.3 (C-3′′); 134.9 (C-1); 131.9 (C-6′); 131.7 (C-7′′); 130.5 (C-9′); 129.1 (C-4); 129.0 (C-2 and C-6); 128.5 (C-3 and C-5); 123.7 (C-8′); 120.7 (C-8); 119.3 (C-2′′); 117.8 (C-3′); 114.4 (C-1′); 103.3 (C-5′); 65.4 (C-1′′); 39.5 (C-4′′); 26.3 (C-5′′); 25.8 (C-11′); 25.7 (C-8′′); 21.8 (C-7′); 17.9 (C-10′); 17.7 (C-10′′); 16.7 (C-9′′). MS: M + H ion *m*/*z* 445.6261 (C_30_H_36_O_3_: 444.6051).

*(2E)-1-[4-(Farnesyloxy)-2-hydroxy-3-(3-methylbut-2-en-1-yl)phenyl]-3-phenylprop-2-en-1-one* (**8**). Yellow solid. Yield: 70.0%, melting point: 62–64 °C. IR (KBr, cm^−1^): 2955, 1632, 1541, 1374, 1203, 1140, 978. ^1^H-NMR (400 MHz, CDCl_3_): δ 13.37 (s, 1H, 2′-OH), 7.87 (d, *J* = 15.5 Hz, 1H, H-8); 7.77 (d, *J* = 9.1 Hz, 1H, H-6′); 7.65 (m, 2H, H-2 and H-6); 7.60 (d, *J* = 15.4 Hz, 1H, H-7); 7.42 (m, 3H, H-3, H-4 and H-5); 6.49 (d, *J* = 9.0 Hz, 1H, H-5′); 5.48 (m, 1H, H-2′′); 5.25 (m, 1H, H-8′); 5.08 (m, 2H, H-6′′ and H-10′′); 4.65 (d, *J* = 6.6 Hz, 2H, H-1′′); 3.39 (d, *J* = 7.2 Hz, 1H, H-7′); 2.09 (m, 4H, H-4′′, H-5′′, H-8′′ and H-9′′); 1.80 (s, 6H, H-10′ and H-13′′); 1.75 (s, 3H, H-11′); 1.67 (s, 6H, H-14′′ and H-15′′). ^13^C-NMR (100 MHz, CDCl_3_): δ 192.1 (C-9); 163.2 (C-2′); 162.9 (C-4′); 144.0 (C-7); 141.3 (C-3′′); 135.3 (C-7′′); 134.9 (C-1); 131.5 (C-9′ and C-11′′); 131,3 (C-6′); 130.5 (C-4); 129.0 (C-2 and C-6); 128.5 (C-3 and C-5); 124.3 (C-6′′); 123.6 (C-10′′); 122.1 (C-8); 120.7 (C-8); 119.3 (C-2′′) 117.9 (C-3′); 114.5 (C-1′); 103.3 (C-5′); 65.4 (C-1′′); 39.7 (C-4′′); 39.5 (C-8′′); 26.7 (C-9′′); 26.2 (C-5′′); 25.8 (C-12′′); 25.7 (C-11′); 21.8 (C-7′); 17.9 (C-10′); 17.7 (C-15′′); 16.7 (C-14′′); 16.0 (C-13′). MS: M + H ion *m*/*z* 513.7351 (C_35_H_44_O_3_: 512.7220).

### 3.5. Oomycete Strain 

Pure strains of *S. parasitica* and *S. australis* were received from the Cell Biology Laboratory, Faculty of medicine, Universidad de Valparaíso, placed on potato dextrose agar (PDA) slants, and stored at 4 °C. This pure strain was isolated from *Salmo salar* carp eggs [33].

### 3.6. Minimum Inhibitory Concentration Evaluation 

The method used in this study for anti-oomycete activity assay was performed according to methods previously reported [34]. The compounds **1**–**8** were tested at 200.0, 150.0, 100.0, 50.0, 25.0, 12.5, 6.3, and 3.1 µg/L to find a preliminary minimum inhibitory concentration (MIC) interval. The MIC values were recorded visually on the basis of mycelia growth. All the independent experiments were conducted three times with quadruplicates at each test concentration. Ethanol solution 1% in water was the negative control and bronopol, clotrimazole, and itraconazole were the positive controls.

### 3.7. Spores Germination Inhibition Assay

The spore germination assay against *Saprolegnia* strains was performed according to the agar dilution method [35]. The minimum oomyceticidal concentration (MOC) and detailed protocols for the biological assays was defined previously [34]. 

### 3.8. Mycelial Growth Inhibition Assay

Inhibition of mycelial growth was assayed using the method described by Hu [29] with small modifications. Oomycete growth was measured as the colony diameter, and toxicity of the compounds **1**–**8** against *Saprolegnia* strains was measured in terms of the percentage of mycelia inhibition by a formula described in detail elsewhere [34]. 

### 3.9. Statistical Analysis 

The statistical data of recovery rates were performed by comparison within isolates and between culturing media following a standard method [20].

## 4. Conclusions

In the current study, we demonstrated that the novel oxyalkylated analogues of isocordoin (**2**–**8**) showed higher inhibitory effect, but isocordoin **1** and chalcone with a geranyl group, such as **7,** showed very weak activity, when compared with bronopol, clotrimazole, and itraconazole. Derricin (**2**) and compound **3** are the main compounds responsible for the observed biological activity against *Saprolegnia* strains and are potential candidates for drug development based on their effective anti-oomycete activities.

## Figures and Tables

**Table 1:** The MIC values ^a^ of the compounds **1**–**8** against *Saprolegnia* species.

Compounds	MIC (µg/mL)	
	*S. parasitica*	*S. australis*
**1**	>200	200
**2**	75	50
**3**	100	75
**4**	125	125
**5**	100	75
**6**	150	125
**7**	>200	>200
**8**	150	75
**Bronopol**	>200	175
**Clotrimazole**	125	150
**Itraconazole**	100	100

^a^ Each value represents the mean ± SD of three experiments (*p* < 0.05), performed in quadruplicate.

**Table 2:** The MOC values ^a^ of the compounds **1**–**8** against spores at 72 h.

Compounds	MOC (µg/mL)	
	*S. parasitica*	*S. australis*
**1**	>200	>200
**2**	75	75
**3**	100	100
**4**	125	125
**5**	125	100
**6**	150	150
**7**	>200	>200
**8**	150	100
**Bronopol**	>200	200
**Clotrimazole**	150	175
**Itraconazole**	100	100

^a^ Each value represents the mean ± SD of three experiments, performed in quadruplicate.

**Table 3:** The MIG values of the compounds **1**–**8** against mycelium at 48 h.

Compounds (200 µg/mL)	MIG (%)	
	*S. parasitica*	*S. australis*
**1**	0	0
**2**	52	58
**3**	38	42
**4**	35	38
**5**	25	30
**6**	28	30
**7**	0	0
**8**	31	33
**Bronopol**	0	33
